# User Local Coordinate-Based Accompanying Robot for Human Natural Movement of Daily Life

**DOI:** 10.3390/s21113889

**Published:** 2021-06-04

**Authors:** Hsiao-Kuan Wu, Po-Yin Chen, Hong-Yi Wu, Chung-Huang Yu

**Affiliations:** 1 Department of Physical Therapy and Assistive Technology, National Yang Ming Chiao Tung University, Taipei 11221, Taiwan; shogunwu@ym.edu.tw (H.-K.W.); pychen1206@gm.ym.edu.tw (P.-Y.C.); hongyiwu@ym.edu.tw (H.-Y.W.); 2 Center for General Education, National Yang Ming Chiao Tung University, Taipei 11221, Taiwan; 3 Department of Physical Medicine and Rehabilitation, Taipei Veterans General Hospital, Taipei 11217, Taiwan; 4 School of Medicine, National Yang-Ming University, Taipei 11221, Taiwan

**Keywords:** accompanying robot, assistive technology, autonomous following in front

## Abstract

Considering the trend of aging societies, accompanying technology can help frail, elderly individuals participate in daily activities. The ideal accompanying robot should accompany the user in a proper position according to the activity scenarios and context; the prerequisite is that the accompanying robot should quickly move to a designated position and closely maintain it regardless of the direction in which the user moves. This paper proposes a user local coordinate-based strategy to satisfy this need. As a proof of concept, a novel “string-pot” approach was utilized to measure the position difference between the robot and the target. We implemented the control strategy and assessed its performance in our gait lab. The results showed that the robot can follow the user in the designated position while the user performs forward, backward, and lateral movements, turning, and walking along a curve.

## 1. Introduction

Many accompanying robots have been developed to help people perform predefined marine, overground, and aeronautical tasks [1,2,3,4,5,6,7,8,9,10,11,12,13,14,15,16,17,18,19,20]. Considering the trend of aging societies, we aim to extend the accompanying technology to help frail, elderly individuals participate in daily activities.

To reduce the physical burden on elderly people, shopping carts, e.g., those proposed in [2], that can automatically follow behind the user have been developed. Other devices such as smart luggage [3,4] and golf carts [5] are available on the market to facilitate certain activities. However, the “following-behind” approach sometimes imposes a psychosocial impact on users, because they cannot see the device and must frequently check whether the device is lost [6,10,13].

To solve this psychosocial problem, researchers have begun to develop robots that are located in front of or to the side of the person while accompanying him or her [6,7,8,9,10]. Front-accompanying robots enable the user to check the robot’s location and access the robot with ease. However, an accompanying robot that is always situated in front would interfere with some of the user’s activities, such as fetching items from a shelf in a supermarket, opening doors, and interacting with friends. Therefore, a robot that can accompany the user in different positions is desirable for daily living activities.

In previous studies [2,7,8,9,14,18,19,20,21] on autonomous following, there was limited discussion on the user’s sideways walking or sharp turning [6,22], which are essential for daily activities. Moreover, it appears that when the user turns, the robot does not move around the user; instead, the user moves around the robot. The reasons include the limited degrees of freedom (DOFs) of the moving platform and the user’s movement tracking strategy. As Hu [7] noted, a user implicitly smoothly moves to cooperate with the accompanying robot movement. In other words, a user reduces the accustomed moving sideways or sharp turns to prevent the robot from failing to follow the user, which also reduces the usability of the accompanying robot in daily activities.

The ideal goal of an accompanying robot for daily living is that the robot can accompany the user in a proper position according to the activity scenarios and context and that the user can move naturally without worry. To achieve this goal, there are two levels of tasks. First, the position at which the robot should accompany the user must be determined. This step can be accomplished by explicit commands from the user [11,14], and the robot can be helped by other techniques such as obstacle avoidance, path planning [10,12,13], or artificial intelligence [15,16,17] in the future. The second and fundamental task is that the robot should quickly move to the designated position and closely maintain it regardless of the direction in which the user moves. This second task is the focus of this study.

The first prerequisite to closely following the user’s movements is that the robot must be as agile as the human, i.e., it should at least be able to move on a plane with 3 DOFs. Many designs, e.g., Mecanum wheels [23], have been proposed and are outside the scope of this study. The other important issue in closely following the user is the tracking method. For example, if the robot tracks the course of the user’s movement, it may not be able to determine whether the user is moving backward or turning around and subsequently moving forward. To date, a single method for a robot to follow a user in any designated position has not been developed. This paper proposes a user local coordinate-based method to satisfy this demand. To prove its concept, we implemented a system and assessed its performance in our gait lab.

## 2. Methods

### 2.1. Concept of the User Local Coordinate-Based Accompanying Method

**Figure 1:**
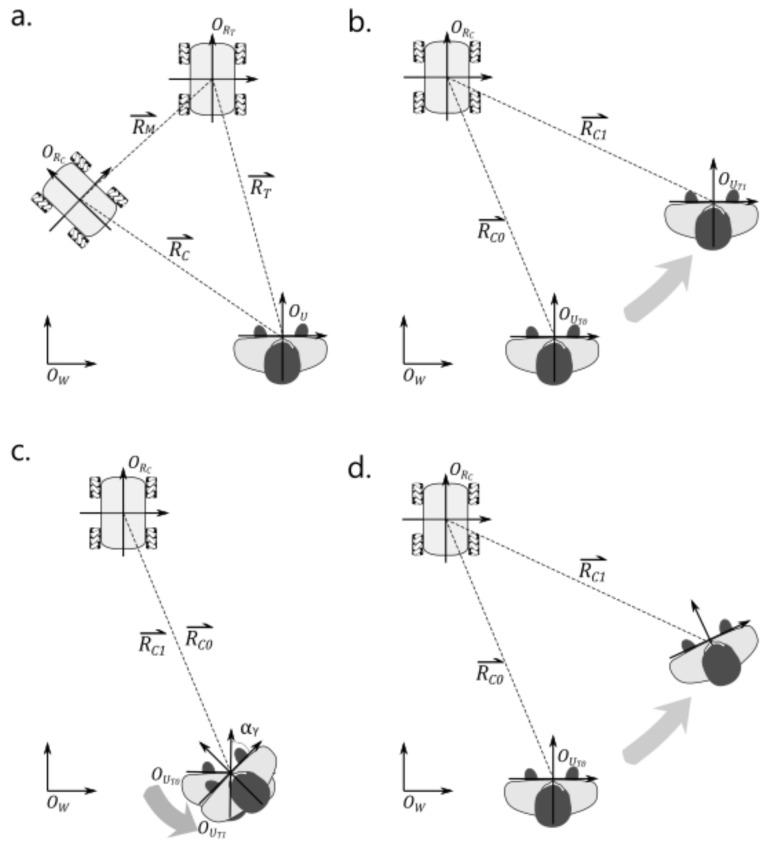
The movements of the accompanying robot and the user. (**a**) the robot translates and rotates to the target position; (**b**) the user translates without rotation; (**c**) the user rotates in place without translation; (**d**) the user translates and rotates.

The concept of the proposed accompanying method is that the robot should move to the specified position with respect to the user’s local coordinate system ($LCS_{U}$)**.** As shown in Figure 1a, the objective is to make $\vec{R_{C}}$ (the current position and orientation of the robot relative to the user) approach $\vec{R_{T}}$ (the target position and orientation relative to the user), i.e., to make the position difference $\Delta \vec{R_{M}}$ approach the zero vector as given in Equation (1). Its underlying tasks include determining the target position, obtaining the robot’s position, calculating the position difference between the target and the accompanying robot in $LCS_{U}$, and moving the robot in the robot coordinate system ($LCS_{R}$) to reduce the position difference. By minimizing the position difference, the robot can closely accompany the user in a designated position while the user moves naturally.

The pseudocode of this algorithm is listed as follows:

1. System Initialize
2. Setup and reset user’s coordinate system
3. Setup and reset robot’s coordinate system
4. Set target position P_t_ of the accompanying robot w.r.t the user
5. Measure the position P_c_ of the accompanying robot w.r.t the user
6. Calculate the errors E_u_ between P_c_ and P_t_
7. Convert E_u_ to E_r_ in the accompanying robot’s local coordinate system
8. Move the robot to reduce the error E_r_ according to a control algorithm
9. Repeat steps 4~8 until system stops following

Because both local coordinate systems of the user ($O_{U}$) and robot ($O_{R}$) are moving and rotating, the coordinate transformation of the position difference between $LCS_{U}$ and $LCS_{R}$ must be clarified. In this study, it was assumed that the target position was predetermined; thus, the required position difference rested on determining the current position of the robot in $LCS_{U}$. The transformation equation is derived as follows:

$$\vec{R_{T}}=\vec{O_{U}O_{RT}}=\left[\begin{array}{ccc} X_{T} & Y_{T} & 1 \end{array}\right]\;\vec{R_{C}}=\vec{O_{U}O_{RC}}=\left[\begin{array}{ccc} X_{C} & Y_{C} & 1 \end{array}\right]$$

Objective: move $O_{RC}$ to $O_{RT}$, i.e.,

(1) $$\Delta \vec{R_{M}}=\vec{R_{T}}-\vec{R_{C}}=\left[\begin{array}{ccc} 0 & 0 & 1 \end{array}\right]$$

where

$$O_{w}:World\;coordinate\;system\;\left(WCS\right)\;O_{U}:User^{\prime }s\;local\;coordinate\;system\;\left(LCS_{U}\right)\;O_{R}:Robot^{\prime }s\;local\;coordinate\;system\;\left(LCS_{R}\right)\;\vec{R_{T}}:Robot^{\prime }s\;target\;position\;relative\;to\;\mathrm{the}\;user\;\vec{R_{C}}:Robot^{\prime }s\;current\;position\;relative\;to\;\mathrm{the}\;user\;\Delta \vec{R_{M}}:Position\;difference$$

For convenience, homogeneous coordinates are used. In general, the user is moving, so $LCS_{U}$ and $\vec{R_{C}}$ in $LCS_{U}$ will change even when the robot is stationary. The three moving scenarios are discussed below.

The first scenario is that the user performs a translation without rotation, as shown in Figure 1b. Assuming the user translates from $O_{U_{T0}}$ to $O_{U_{T1}}$, let $\vec{O_{U_{T0}}O_{U_{T1}}}=\left[\begin{array}{cc} \Delta X_{t} & \Delta Y_{t} \end{array}\right]$ in the world coordinate system $\left(WCS\right)$, and view the movement from the perspective of $LCS_{U}$.

(2) $$\begin{array}{ll} \vec{R_{C1}}=\vec{O_{U_{T1}}O_{R_{C0}}} & =\vec{R_{C0}}\left[\begin{array}{ccc} 1 & 0 & 0\\ 0 & 1 & 0\\ -\Delta X_{t} & -\Delta Y_{t} & 1 \end{array}\right]\\ & ={\left[\begin{array}{c} X_{C0}\\ Y_{C0}\\ 1 \end{array}\right]}^{T}-{\left[\begin{array}{c} \Delta X_{t}\\ \Delta Y_{t}\\ 1 \end{array}\right]}^{T} \end{array}$$

The second scenario is that the user performs a rotation in place without translation, as shown in Figure 1c. $\vec{O_{U_{T0}}O_{U_{T1}}}$ rotates with $\alpha_{r}$ in WCS and is viewed from $LCS_{U}$.

(3) $$\begin{array}{l} \vec{R_{C1}}=\vec{O_{U_{T1}}O_{R_{C1}}}=\vec{O_{U_{T0}}O_{R_{C0}}}\;\mathrm{rotate}\;-\alpha_{r}\\ =\vec{R_{C0}}\left[\begin{array}{ccc} \cos\left(-\alpha_{r}\right) & \sin\left(-\alpha_{r}\right) & 0\\ -\sin\left(-\alpha_{r}\right) & \cos\left(-\alpha_{r}\right) & 0\\ 0 & 0 & 1 \end{array}\right]\\ ={\left[\begin{array}{c} X_{C0}\\ Y_{C0}\\ 1 \end{array}\right]}^{T}\left[-\begin{array}{ccc} \cos\left(-\alpha_{r}\right) & \sin\left(-\alpha_{r}\right) & 0\\ \sin\left(-\alpha_{r}\right) & \cos\left(-\alpha_{r}\right) & 0\\ 0 & 0 & 1 \end{array}\right]={\left[\begin{array}{c} X_{C0}\cos\alpha_{r}+Y_{C0}\sin\alpha_{r}\\ -X_{C0}\sin\alpha_{r}+Y_{C0}\cos\alpha_{r}\\ 1 \end{array}\right]}^{T} \end{array}$$

$LCS_{R}$ is also rotated by $\alpha_{r}$.

The third scenario is that the user translates and rotates, as shown in Figure 1d; then,

(4) $$\begin{array}{l} \vec{R_{C1}}=\vec{R_{C0}}\left[\begin{array}{ccc} \cos\left(-\alpha_{r}\right) & \sin\left(-\alpha_{r}\right) & 0\\ -\sin\left(-\alpha_{r}\right) & \cos\left(-\alpha_{r}\right) & 0\\ -\Delta X_{t} & -\Delta Y_{t} & 1 \end{array}\right]\\ ={\left[\begin{array}{c} X_{C0}\\ Y_{C0}\\ 1 \end{array}\right]}^{T}\left[\begin{array}{ccc} \cos\left(-\alpha_{r}\right) & \sin\left(-\alpha_{r}\right) & 0\\ -\sin\left(-\alpha_{r}\right) & \cos\left(-\alpha_{r}\right) & 0\\ -\Delta X_{t} & -\Delta Y_{t} & 1 \end{array}\right]\\ ={\left[\begin{array}{c} X_{C0}\\ Y_{C0}\\ 1 \end{array}\right]}^{T}\left[\begin{array}{ccc} \cos\left(-\alpha_{r}\right) & \sin\left(-\alpha_{r}\right) & 0\\ -\sin\left(-\alpha_{r}\right) & \cos\left(-\alpha_{r}\right) & 0\\ 0 & 0 & 1 \end{array}\right]-{\left[\begin{array}{c} \Delta X_{t}\\ \Delta Y_{t}\\ 1 \end{array}\right]}^{T} \end{array}$$

The third scenario is the general case. Combined with Equation (1), this leads to $\Delta \vec{R_{MU}}$, and the position difference in $LCS_{U}$ is

(5) $$\begin{array}{ll} \Delta \vec{R_{MU}}={\left[\begin{array}{c} \Delta X_{U}\\ \Delta Y_{U}\\ 1 \end{array}\right]}^{T} & =\vec{R_{T}}-\vec{R_{C1}}\\ & ={\left[\begin{array}{c} X_{T}\\ Y_{T}\\ 1 \end{array}\right]}^{T}-\left({\left[\begin{array}{c} X_{C0}\\ Y_{C0}\\ 1 \end{array}\right]}^{T}\left[\begin{array}{ccc} \cos\left(-\alpha_{r}\right) & \sin\left(-\alpha_{r}\right) & 0\\ -\sin\left(-\alpha_{r}\right) & \cos\left(-\alpha_{r}\right) & 0\\ 0 & 0 & 1 \end{array}\right]-{\left[\begin{array}{c} \Delta X_{t}\\ \Delta Y_{t}\\ 1 \end{array}\right]}^{T}\right) \end{array}$$

$LCS_{R}$ is also rotated by $\alpha_{r}$.

In implementation, the robot moves in $LCS_{R}$; therefore, $\Delta \vec{R_{MU}}$ should further rotate with $\left(\gamma_{C0}\right)$ and yield $\Delta \vec{R_{MR}}$ as the position difference in $LCS_{R}$.

(6) $$\begin{array}{ll} \Delta \vec{R_{MR}}={\left[\begin{array}{c} \Delta X_{R}\\ \Delta Y_{R}\\ 1 \end{array}\right]}^{T} & ={\left[\begin{array}{c} \Delta X_{U}\\ \Delta Y_{U}\\ 1 \end{array}\right]}^{T}\left[\begin{array}{ccc} \cos\left(-\gamma_{C0}\right) & \sin\left(-\gamma_{C0}\right) & 0\\ -\sin\left(-\gamma_{C0}\right) & \cos\left(-\gamma_{C0}\right) & 0\\ 0 & 0 & 1 \end{array}\right]\\ & ={\left[\begin{array}{c} X_{T}\\ Y_{T}\\ 1 \end{array}\right]}^{T}\left[\begin{array}{ccc} \cos\left(\gamma_{C0}\right) & -\sin\left(\gamma_{C0}\right) & 0\\ \sin\left(\gamma_{C0}\right) & \cos\left(\gamma_{C0}\right) & 0\\ 0 & 0 & 1 \end{array}\right]\\ & -{\left[\begin{array}{c} X_{C0}\\ Y_{C0}\\ 1 \end{array}\right]}^{T}\left[\begin{array}{ccc} \cos\left(\alpha_{r}+\gamma_{C0}\right) & -\sin\left(\alpha_{r}+\gamma_{C0}\right) & 0\\ \sin\left(\alpha_{r}+\gamma_{C0}\right) & \cos\left(\alpha_{r}+\gamma_{C0}\right) & 0\\ 0 & 0 & 1 \end{array}\right]\\ & +{\left[\begin{array}{c} \Delta X_{t}\\ \Delta Y_{t}\\ 1 \end{array}\right]}^{T}\left[\begin{array}{ccc} \cos\left(\alpha_{r}+\gamma_{C0}\right) & -\sin\left(\alpha_{r}+\gamma_{C0}\right) & 0\\ \sin\left(\alpha_{r}+\gamma_{C0}\right) & \cos\left(\alpha_{r}+\gamma_{C0}\right) & 0\\ 0 & 0 & 1 \end{array}\right] \end{array}$$

With the error between the robot’s position and the target position, i.e., $\mathrm{Equation}\;\left(6\right)$, and by applying a control law, e.g., PID, to make $\vec{R_{MR}}\to \vec{0}$, the robot can accompany the user in any position, including in front, and in any movement direction, including forward, backward, sideways, rotating in place, etc. To maintain the orientation of the robot in $LCS_{U}$, the robot should also rotate by $\alpha_{r}$.

### 2.2. Embodiment of the Accompanying Robot with the $LCS_{U}$ Viewpoint

**Figure 2:**
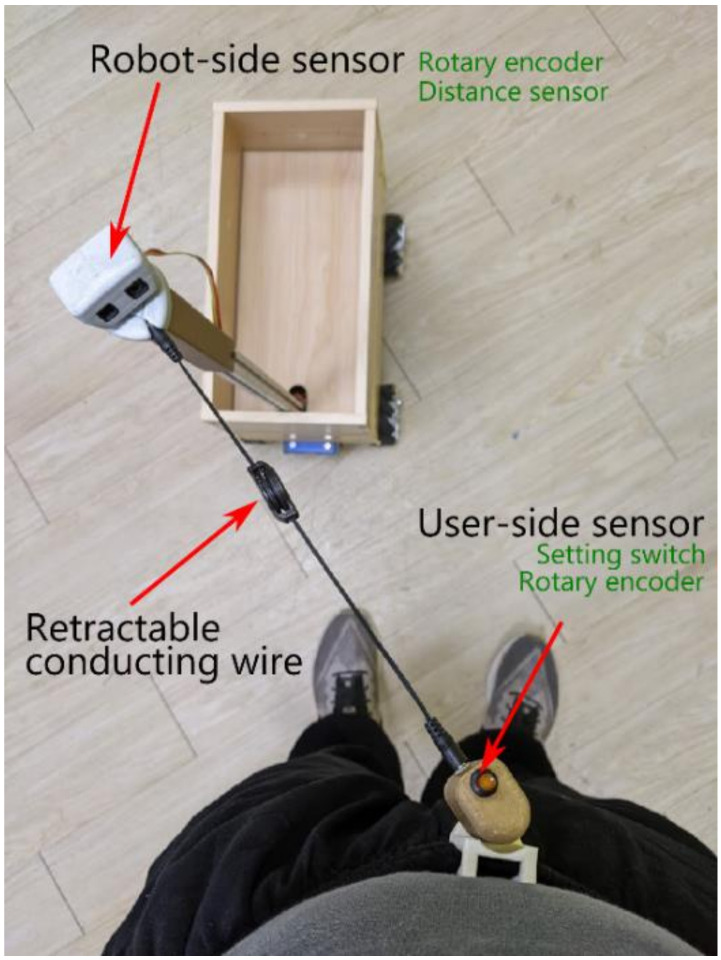
“String-pots” to sense $LCS_{U}$ and $LCS_{R}$.

To measure the position difference between the robot and the target, a novel “string-pot” approach was utilized. It is composed of two rotary encoders, one distance sensor, and a retractable conducting wire, as shown in Figure 2. The shafts of the two rotary encoders are parallel to each other and fixed on the user and the robot. The distance sensor is attached to the housing of the robot’s encoder. The retractable wire connects the housing of the encoders, pulls them to face each other, and ensures that the distance sensor always points to the user. In addition, the retractable conducting wire can transmit the encoder’s signal from the user to the robot. This arrangement can easily measure the orientations of $LCS_{U}$ and $LCS_{R}$ with respect to each other and the distance between them. With this measurement system, $\vec{R_{c1}}$ is directly measured w.r.t. $LCS_{U}$, and Equations (4) and (6) become Equations (7) and (8), respectively.

(7) $$\vec{R_{C1}}={\left[\begin{array}{c} d\cdot \cos\left(-\theta_{U}\right)\\ d\cdot \sin\left(-\theta_{U}\right)\\ 1 \end{array}\right]}^{T}$$

(8) $$\begin{array}{l} \Delta \vec{R_{MR}}={\left[\begin{array}{c} \Delta X_{R}\\ \Delta Y_{R}\\ 1 \end{array}\right]}^{T}={\left[\begin{array}{c} \Delta X_{U}\\ \Delta Y_{U}\\ 1 \end{array}\right]}^{T}\left[\begin{array}{ccc} \cos\left(\theta_{U}+\theta_{R}\right) & \sin\left(\theta_{U}+\theta_{R}\right) & 0\\ -\sin\left(\theta_{U}+\theta_{R}\right) & \cos\left(\theta_{U}+\theta_{R}\right) & 0\\ 0 & 0 & 1 \end{array}\right]\\ =\left({\left[\begin{array}{c} X_{T}\\ Y_{T}\\ 1 \end{array}\right]}^{T}-{\left[\begin{array}{c} d\cdot \cos\left(-\theta_{U}\right)\\ d\cdot \sin\left(-\theta_{U}\right)\\ 1 \end{array}\right]}^{T}\right)\;\left[\begin{array}{ccc} \cos\left(\theta_{U}+\theta_{R}\right) & \sin\left(\theta_{U}+\theta_{R}\right) & 0\\ -\sin\left(\theta_{U}+\theta_{R}\right) & \cos\left(\theta_{U}+\theta_{R}\right) & 0\\ 0 & 0 & 1 \end{array}\right] \end{array}$$

where

$$d:\;distance\;between\;the\;user\;and\;the\;robot\;\theta_{U\;}:encoder\;value\;on\;the\;user\;\theta_{R\;}:encoder\;value\;on\;the\;robot$$

The measured encoder values are negative for the user orientation change; i.e.,

$$\Delta \vec{R_{MR}}\theta =\theta_{U}+\theta_{R}$$

where

$\Delta \vec{R_{MR}}\theta \;\mathrm{is}\;\mathrm{the}\;\mathrm{rotation}\;\mathrm{angle}\;\mathrm{of}\;LCS_{R}$ to maintain the orientation w.r.t. $LCS_{U}$

As mentioned, while performing daily activities, humans utilize all 3 DOFs, including moving forward, backward, and sideways and turning in place. To simplify the movement control, we utilized Mecanum wheels [23] for the 3-DOF moving platform.

**Figure 3:**
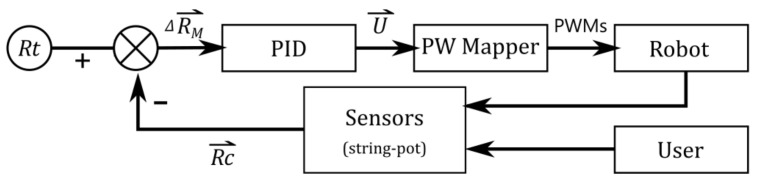
Control system structure.

The control system structure is shown in Figure 3. A PID controller was implemented, and $\Delta \vec{R_{MR}}$ was used as its error input. The output of the PID controller was the velocity command $\left[\begin{array}{ccc} V_{x} & V_{y} & \omega_{z} \end{array}\right]$ for the robot to execute, as shown in Equation (9).

(9) $$\Delta \vec{R_{MR}}+\Delta \vec{R_{MR}}\theta ={\left[\begin{array}{c} \Delta x_{R}\\ \Delta y_{R}\\ \Delta \theta_{R} \end{array}\right]}^{T}\overset{\mathrm{PID}}{\Rightarrow }\vec{U}={\left[\begin{array}{c} V_{x}\\ V_{y}\\ \omega_{z} \end{array}\right]}^{T}$$

Since the four Mecanum wheels were used, the individual rotational speeds of the wheels could be calculated for the intended movement, and the pulse-width modulation (PWM) signals could be converted for their motor drivers using the pulse-width (PW) mapper as shown in Equation (10) [24].

(10) $$\mathrm{PW}\;\mathrm{mapper}:\left[\begin{array}{c} PWM_{1}\\ PWM_{2}\\ \begin{array}{c} PWM_{3}\\ PWM_{4} \end{array} \end{array}\right]=k\left[\begin{array}{c} \omega_{1}\\ -\omega_{2}\\ \begin{array}{c} \omega_{3}\\ -\omega_{4} \end{array} \end{array}\right]=\frac{k}{R}\;\left[\begin{array}{c} \begin{array}{ccc} +1 & +1 & \left(L_{1}+L_{2}\right) \end{array}\\ \begin{array}{ccc} +1 & -1 & \left(L_{1}+L_{2}\right) \end{array}\\ \begin{array}{c} \begin{array}{ccc} -1 & +1 & \left(L_{1}+L_{2}\right) \end{array}\\ -\begin{array}{ccc} 1 & -1 & \left(L_{1}+L_{2}\right) \end{array} \end{array} \end{array}\right]\left[\begin{array}{c} V_{x}\\ V_{y}\\ \omega_{z} \end{array}\right]$$

where

$V_{x},\;V_{y},\;\omega_{z}$: velocity of the robot
$\omega_{1},\;\omega_{2},\;\omega_{3,}\omega_{4}$: angular velocity of the four Mecanum wheels
R: radius of the Mecanum wheels
k: motor constant
PWM: pulse width modulation

### 2.3. Detailed System Hardware

**Figure 4:**
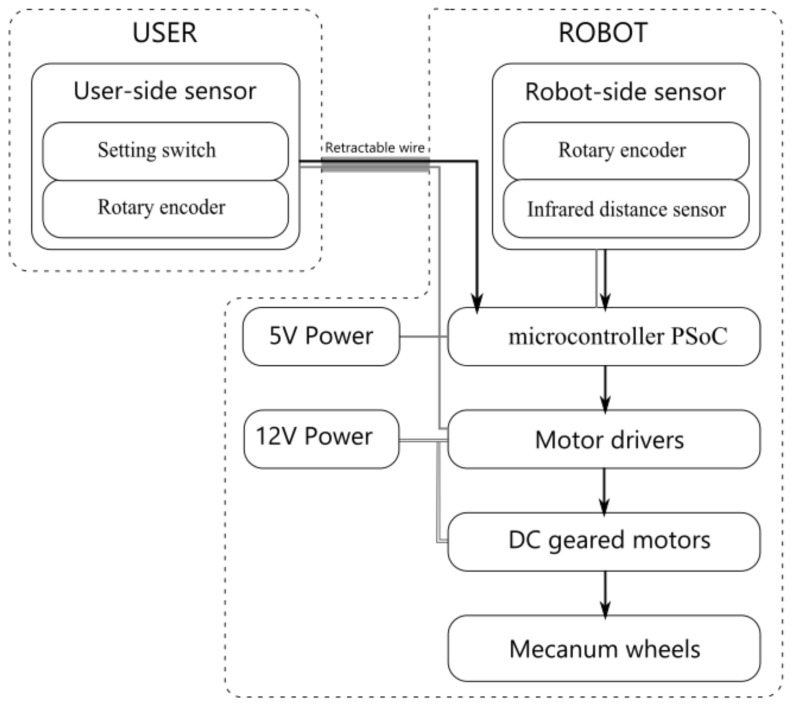
System structure block diagram.

**Figure 5:**
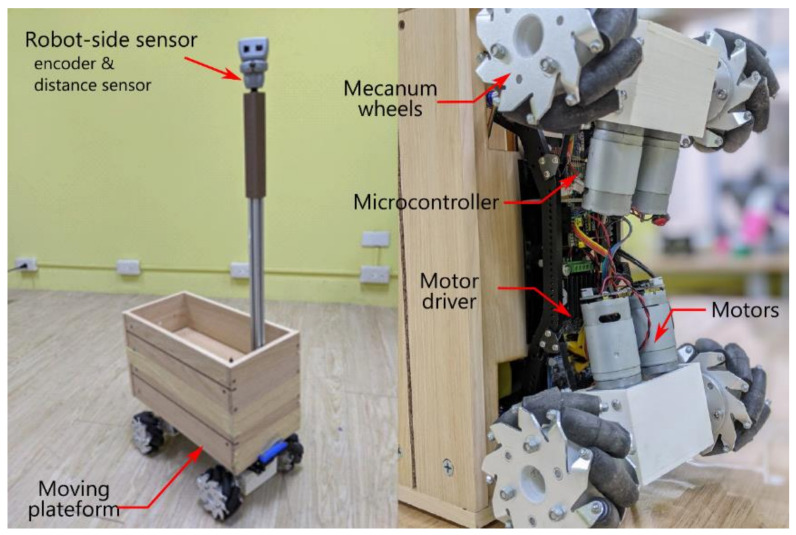
Assembly of the tested accompanying robot.

The string-pot system in Figure 2 was realized with an infrared distance sensor (SHARP GP2Y0A02YK0F) and two absolute rotary encoders (P3015 Series Hall Rotary Encoder). Their signals were acquired by a programmable system-on-chip (PSoC) microcontroller (Cypress, CY8CKIT-059 PSoC 5 LP prototyping kit) and a built-in 12-bit analog-to-digital converter (ADC) with a sampling rate of 2000 samples per second (s/s). The data were filtered with a median filter and subsequently downsampled to 50 s/s to calculate the position difference $\Delta \vec{R_{MR}}$. Then, the position difference was further converted to a pulse width through a PID controller and a PW mapper. Through the built-in PWM module of the PSoC, the pulse widths were transmitted to the motor drivers (DC 5–12 V 0–30 A Dual-channel H Bridge Motor Driver Controller) to drive the DC geared motor (GW4058-31ZY DC worm gear motor) and Mecanum wheels. The system structure block diagram was shown in Figure 4. The appearance of the tested accompanying robot was shown in Figure 5.

## 3. Methods of System Verification

### 3.1. Testing Tasks

To assess the performance of the proposed accompanying method, six basic walking tasks and two types of combined movement were performed. The six basic walking tasks were: forward walking, backward walking, left lateral walking, right lateral walking, a left pivot of 90°, and a right pivot of 90°. These tasks were performed with a 2.5 m × 1.8 m rectangle of the force plate border as a reference. The two types of combined maneuvers were (1) walking along the force plate while the user maintained a front-facing orientation and (2) walking around the force plate clockwise or counterclockwise. The border of the force plate was used as the walking reference. In addition, the user arbitrarily set or selected the target position. Although the robot’s performance should be identical with different accompanying positions, it was purposely set in front of the user to demonstrate our proposed method capability.

### 3.2. Testing Environment

The tests were performed in our motion lab, which was equipped with an 8-camera VICON motion capture system (VICON Motion Systems, Oxford, UK). Four markers were placed on the user’s pelvis and bilateral anterior and posterior iliac spine (ASIS and PSIS) to calculate the user’s orientation with respect to the lab’s coordinates; four markers were placed on the shelf above the four Mecanum wheels of the robot to calculate the orientation of the robot with respect to the lab’s coordinates. Two other markers were placed on the housing of the user-end and robot-end rotary encoders as the origins of $LCS_{U}$ and $LCS_{R}$, respectively. The origins were not at the centers of the user and robot.

The data from the VICON system were processed to obtain the trajectory of the user and robot by LabVIEW to calculate the orientation of the user and robot, distance between the origins of $LCS_{U}$ and $LCS_{R}$, and relative orientations of $LCS_{R}$ and $LCS_{U}$ with respect to each other. The data were also used to calculate the robot’s trajectory in $LCS_{U}$.

## 4. Results of System Verification

Table 1 lists the positions and displacements of the user and robot in the WCS. The origin of the WCS was set at the user’s start position. The direction and orientation of the WCS were determined with respect to the lab coordinates. First, the user faced the positive Y axis. The robot’s start position was the setting position of the WCS. The displacement was the stop position subtracted from the start position. The movement displacements of the user and robot in the Y direction were 1914 mm and 1933 mm in the forward-walking task, respectively. The movement displacements of the user and robot in the Y direction were −1980 mm and −1985 mm in the backward-walking task, respectively. The displacements of the user and robot in the X direction were −1520 mm and −1732 mm in the left lateral walking task, respectively. The displacements of the user and robot in the X direction were 1558 mm and 1666 mm in the right lateral walking task, respectively. The turning angles of the user and robot changed by 73.7 degrees and 71.6 degrees in the left pivot turning task, respectively. The turning angles of the user and robot changed by −92.1 degrees and −88.4 degrees in the right pivot turning task, respectively.

Table 2 lists the robot’s start, target, and stop positions in $LCS_{U}$. The stop position is defined as the point where the user stopped walking and the robot settled. $X_{C}$ is the robot position w.r.t. $LCS_{U}$ in the lateral direction, $Y_{C}$ is the robot position w.r.t. $LCS_{U}$ in the front direction, and $\gamma_{C}$ is the robot’s orientation w.r.t. $LCS_{U}$.

### 4.1. Basic Walking Tasks

**Figure 6:**
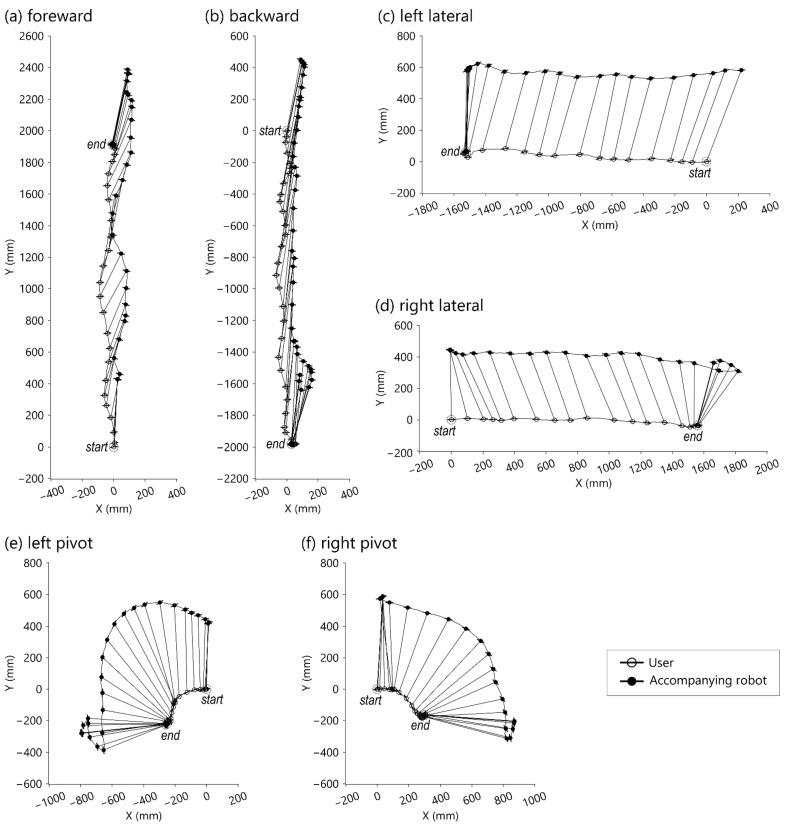
Top-view motion graph of the movements of the robot and user w.r.t. the WCS. (**a**) Forward walking test; (**b**) backward walking test; (**c**) left lateral walking test; (**d**) right lateral walking test; (**e**) walking test with pivot turning to the left; (**f**) walking test with pivot turning to the right.

In the basic walking tasks, the user was asked to perform the tasks inside the rectangular force plate in the center of the motion lab. Figure 6 shows the trajectories of the walking tasks w.r.t. the WCS, where the corresponding origins of $LCS_{U}$ (symbolized ‘○’) and $LCS_{R}$ (symbolized ‘●’) are linked by line segments. The protruding hairline symbols represent medial-lateral directions. The interval between consecutive corresponding point pairs was 200 milliseconds. Lags and overshoots were observed at the beginning and end, respectively. The target/starting position was arbitrarily selected by the user at the beginning of each test. Figure 6a shows the result of the user walking forward 6 steps. Figure 6b shows the result of the user walking backward 8 steps. Figure 6c shows the result of the user performing left lateral walking for 8 steps. Figure 6d shows the result of the user performing right lateral walking for 8 steps.

**Figure 7:**
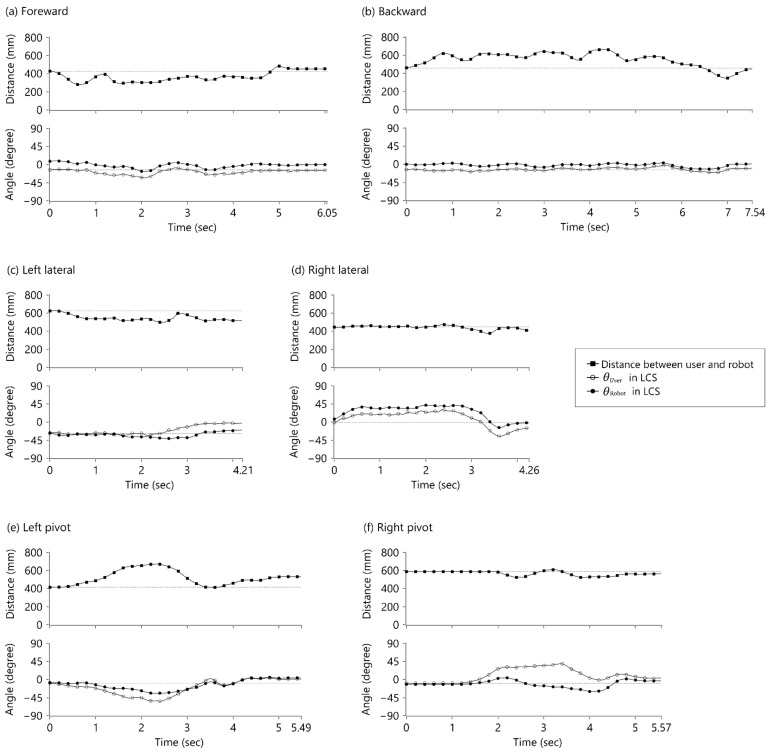
Relative distance between the robot and the user w.r.t. the LCS and encoder values $\theta_{Robot}$, $\theta_{User}$ in the LCS. (**a**) Forward walking test; (**b**) backward walking test; (**c**) left lateral walking test; (**d**) right lateral walking test; (**e**) walking test with pivot turning to the left; (**f**) walking test with pivot turning to the right.

Figure 7 shows the time series of the sensing values for the walking tasks that correspond to the distance between the robot and the user and the encoder values $\theta_{Robot}$, $\theta_{User}$. The gaps between the set distance and actual distance and between $\theta_{User}$ and the target angle represent the lag and overshoot of the track. Lag tracking can be demonstrated in the increased gap between $\theta_{User}$ and $\theta_{Robot}$. The distance between the user and the robot decreased while the user walked forward and increased while the user walked backward due to the phase lag.

### 4.2. Combined Tasks

#### 4.2.1. Walking along a Rectangle

**Figure 8:**
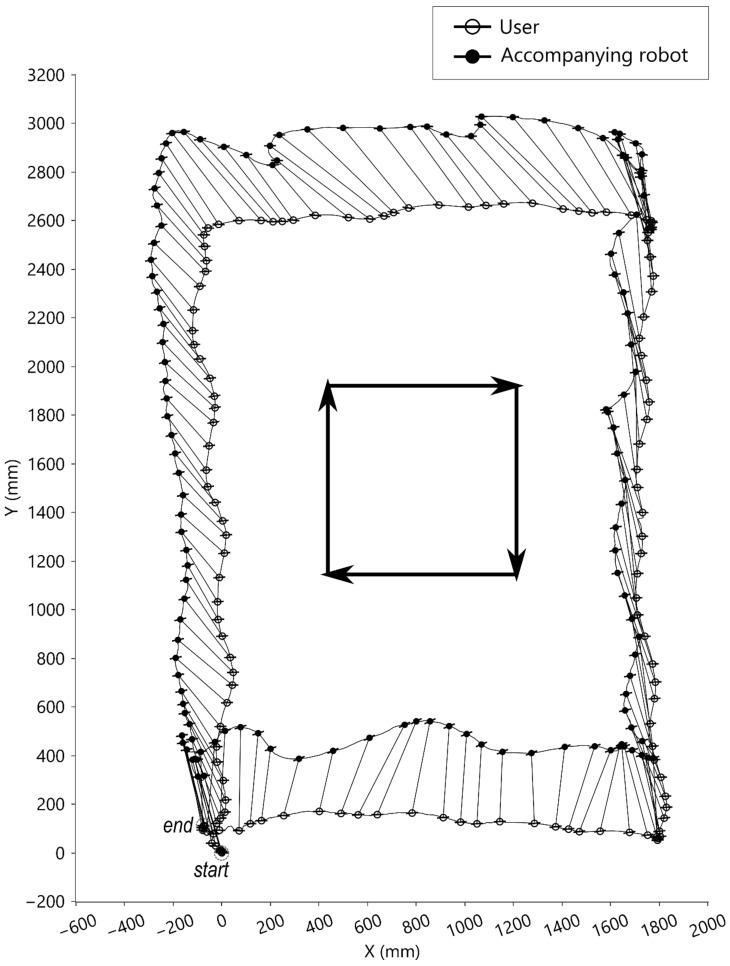
Top-view motion graph of walking along the rectangle.

In the condition of walking along a rectangle, the user was instructed to walk along the rectangular force plate in the center of the motion lab. The movements along the rectangle in this condition included walking forward, laterally right, backward, and laterally left with eight steps per direction. The results are presented in Figure 8. The accompanying robot could follow the user walking along the rectangle. Shown are the trajectories of the walking tasks where the corresponding origins of $LCS_{U}$ (symbolized ‘○’) and $LCS_{R}$ (symbolized ‘●’) are linked by line segments. The protruding hairline symbols represent the medial-lateral directions.

#### 4.2.2. Clockwise-Curve Walking Test

**Figure 9:**
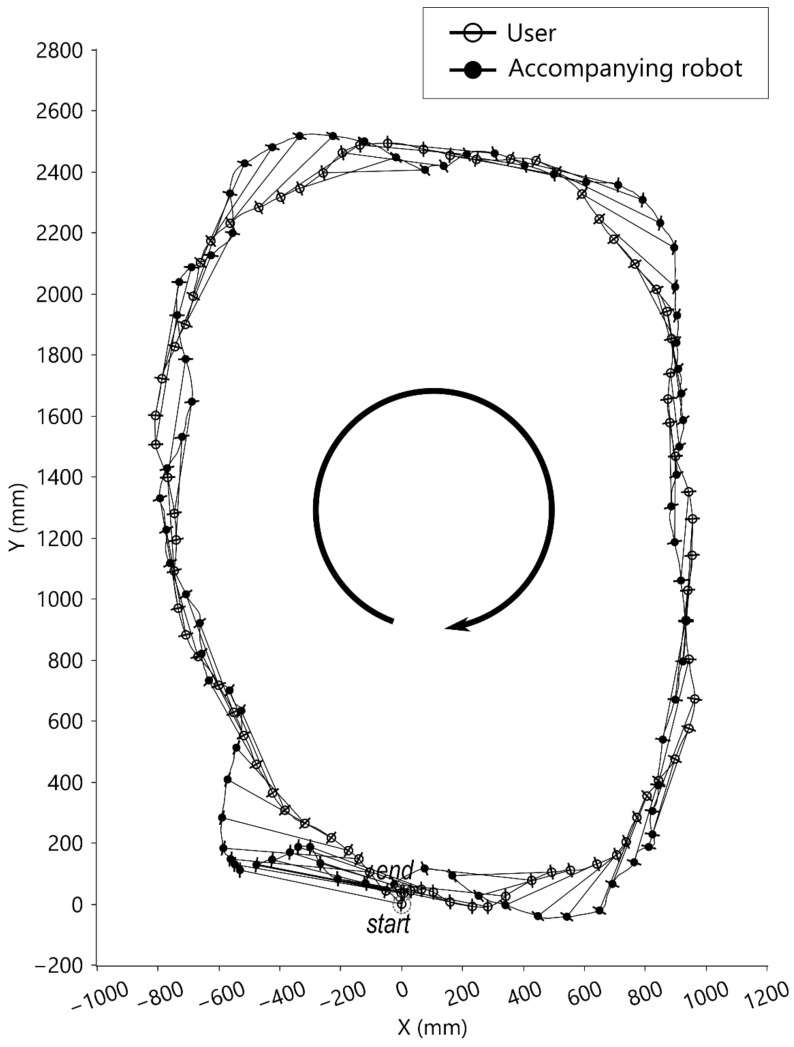
Top-view motion graph of clockwise walking.

In the clockwise-curve walking test, the user was instructed to walk in a circle around the force plate clockwise, which combined forward walking and small right lateral and right turning movements. As shown in Figure 9, the starting position of the robot was on the front right side of the user, and the trajectory was almost on the user’s walking curve. Shown are the trajectories of the walking tasks where the corresponding origins of $LCS_{U}$ (symbolized ‘○’) and $LCS_{R}$ (symbolized ‘●’) are linked by line segments. The protruding hairline symbols represent medial-lateral directions. The total walking steps were 23 steps in this clockwise-curve walking test.

#### 4.2.3. Counterclockwise-Curve Walking Test

**Figure 10:**
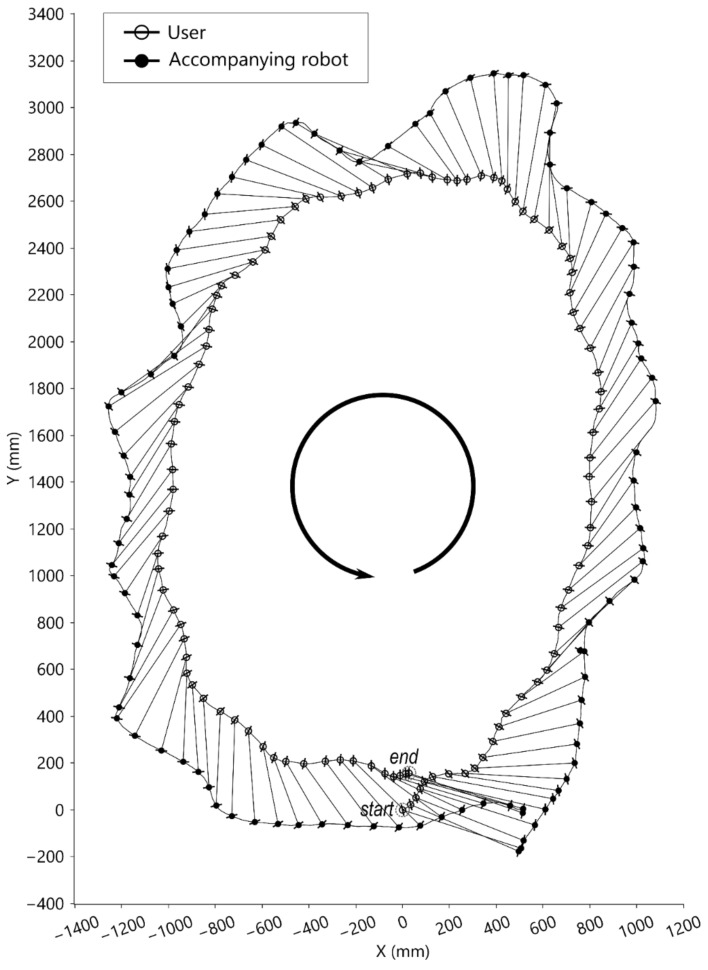
Top-view motion graph of counterclockwise walking.

In the counterclockwise-curve walking test, the user was instructed to walk in a circle around the force plate counterclockwise, which combined forward, left lateral, and left turning movements. As shown in Figure 10, the starting position of the robot was on the front right side of the user. When the user walked counterclockwise, the trajectory of the robot enclosed the trajectory of the user. Shown are the trajectories of the walking tasks where the corresponding origins of $LCS_{U}$ (symbolized ‘○’) and $LCS_{R}$ (symbolized ‘●’) are linked by line segments. The protruding hairline symbols represent the medial-lateral directions. There were 28 walking steps in total in this counterclockwise-curve walking test.

## 5. Discussion

The results show that the robot can follow the user in the designated position while the user performs forward, backward, and lateral movements, turning, and curve walking. In other words, this study has demonstrated that with the proposed tracking strategy, the accompanying robot can closely follow the user in sideways or sharp turn movements. In addition, it can accompany the user in front even when the user moves backwards.

Each test had different start/target positions, which were arbitrarily set. Regardless, the difference between the end position and the set/target position in each test was within an acceptable range for daily accompanying. There were phase lags and overshoots at the end of the tests. Further tuning the PID controller or replacing it with other control algorithms could improve the tracking performance. For example, there appeared to be a small phase lag and no overshoot in the left lateral walking test (Figure 6c and Table 1). After checking the data, we found that the robot was further to the right of the user, and the distance sensor measured the user’s forearm instead of the trunk when setting the target position. In other words, unlike the other tests, in the left lateral walking test, the target distance was shorter than the actual distance between the robot and the user from the beginning. This mistake suggests a control method where changing the target distance may reduce the phase lag and overshoot, i.e., when the user is approaching the robot, e.g., the robot is in front of the user and the user is walking forward, the target distance can be set to a greater value; when the user is moving away from the robot, the target distance can be set shorter. Further control techniques, including system identification, modeling, and simulation, to improve system performance and stability should be examined in the future.

In this study, the selected distance sensor (SHARP GP2Y0A02YK0F) is less affected by environmental light. However, the sensing range was limited from 20 cm to 150 cm, and some measuring errors may occur when the user walks too close to the robot. The absolute rotary encoder (P3015 series hall rotary encoder) is less affected by friction. However, the retractable conducting wire may swing, and the user can rotate his/her body while moving. Consequently, the sensor may not always measure the same point of the user. The trajectory figures (Figure 6, Figure 8, Figure 9 and Figure 10) show that there were natural rotations and sideways movements of the user’s pelvis; thus, the user’s coordinates were not steady, which caused fluctuating target positions in the WCS. There were three possible solutions to remove unnecessary fluctuations in the robot: (1) use a laser range finder; (2) reduce the weight of the retractable wire or become wireless; and (3) reduce the loop rate of the controller. However, a low loop rate can compromise the trajectory tracking performance. Adding a movement filter to smoothen the motion may be better.

In addition to the controller and transducers, the mechanical design of the robot affects the tracking performance. First, the inertia and driving torque of the robot affect the acceleration. Actuator saturation is experienced when the user moves too fast. It can be solved by using high-torque motors or by an algorithm [25]. Driving motors with higher torque can respond more quickly to the user’s sudden movements, but consume more power and increase system weight. Second, the 3 DOFs of the moving platform simplify the control strategy and save time and space in tracking the target position. In this study, this aspect was implemented with Mecanum wheels [23], but other biomimetic techniques [21,26] or unmanned aerial vehicles [22,27] can also be used.

The key point of the proposal was determining the positions of the user and robot w.r.t to each other. We achieved this task using the novel “string-pot” sensor system. It is compact, reliable, and immune to environmental noise such as light. However, with this “string-pot” sensor system, the user-side sensor is not suitable to be worn on the lateral side of the user’s body because the sensor and wire will interfere with the user’s hand. To remove the wire, i.e., create a wireless system, further challenges must be overcome, such as target user identification and user orientation determination. Facial recognition [28] may solve part of this problem, but issues such as the user looking around without changing the body’s position while the robot is following behind must be considered. Obtaining the relative distances and orientations of the user and robot without physical wires is possible. Two inertial measurement units (IMUs) [18,19], mounted on both user side and robot side, and a laser range finder [20] can provide information on the distance and orientation between the robot and the user.

The excursion trajectories of the robot are greatly affected by the target position and user movement tracking. As shown in Figure 6e,f, the robot took a longer path while the user pivoted. Furthermore, as shown in Figure 9 and Figure 10, the user performed similar lengths of circular walks, but the robot trajectories were different. One of the robot’s trajectories was similar to the user’s trajectory, and the other of the robot’s trajectories was larger than the user’s trajectory. The reason was that one of the robot’s target positions was on the walking curve/circle, and the other was outside of the walking curve/circle. If the target point is outside of the instance center’s circle of the user’s moving curve, the robot is expected to take a longer path than the user.

## 6. Conclusions

We have demonstrated that a robot with one algorithm and simple controller can accompany the user in a selected position while the user performs forward, backward, and sideways or sharp turn movements. i.e., the user can move naturally, and the robot can maintain a designed position w.r.t. the user. This goal was achieved using three main factors: (1) knowledge of the positions of the user and robot w.r.t their local coordinate systems; (2) agile movement of the robot; and (3) a quickly updating feedback loop. In the future, using wireless sensors that combine the obstacle avoidance technique and a high-level method to determine the set position of the robot in $LCS_{U}$, the accompanying robot can further help the user in daily life activities.

## Figures and Tables

**Table 1:** Result of Walking Tasks in the WCS.

Tests	Start Position			Stop Position			Displacement		
	$X\;\left(\mathrm{mm}\right)$	$Y\;\left(\mathrm{mm}\right)$	$\gamma \;\left({}^{\circ}\right)$	$X\;\left(\mathrm{mm}\right)$	$Y\;\left(\mathrm{mm}\right)$	$\gamma \;\left({}^{\circ}\right)$	$X\;\left(\mathrm{mm}\right)$	$Y\;\left(\mathrm{mm}\right)$	$\gamma \;\left({}^{\circ}\right)$
Forward walking									
user	0	0	99.6	−3	1914	91.8	−3	1914	−7.8
robot	24	438	78.1	90	2361	77.9	66	1933	−0.2
Backward walking									
user	0	0	92.4	28	−1980	92.8	28	−1980	0.4
robot	88	451	78.8	82	−1534	81.9	−6	−1985	3.1
Left lateral walking									
user	0	0	97.4	−1520	64	92.0	−1520	64	−5.4
robot	220	583	95.7	−1512	582	108.4	−1732	−2	12.7
Right lateral walking									
user	0	0	92	1558	−34	90.4	1558	−34	−1.8
robot	−8	447	83.8	1658	363	77.9	1666	−84	−5.9
Left pivot turning									
user	0	0	97.2	−265	−222	170.9	−266	−222	73.7
robot	10	417	95.9	−796	−279	167.5	−807	−696	71.6
Right pivot turning									
user	0	0	95.5	309	−165	3.4	309	−165	−92.1
robot	34	588	98.6	871	−209	10.1	837	−797	−88.4

**Table 2:** Result of Walking Tasks in $LCS_{U}$.

Tests	Target/Start Position			Stop Position			Average Position during Walking		
	$X_{C}$ (mm)	$Y_{C}$ (mm)	$\gamma_{C}$ (°)	$X_{C}$ (mm)	$Y_{C}$ (mm)	$\gamma_{C}$ (°)	$X_{C}$ (mm)	$Y_{C}$ (mm)	$\gamma_{C}$ (°)
Forward walking	95	418	−12.5	107	443	−13.6	111 ± 33	320 ± 40	−19.3 ± 6.5
Backward walking	106	447	−13.2	75	443	−9.7	128 ± 32	574 ± 44	−12.5 ± 3.1
Left lateral walking	292	550	−28.0	26	517	−2.9	259 ± 31	482 ± 36	−28.3 ± 3.3
Right lateral walking	9	447	−0.7	101	397	−14.3	−159 ± 50	422 ± 13	20.7 ± 6.5
Left pivot turning	63	412	−8.8	−9	533	1.0	310 ± 167	440 ± 26	−32.6 ± 14.9
Right pivot turning	91	582	−8.8	−36	563	3.7	−106 ± 189	536 ± 54	11.5 ± 19.7
Walking along a rectangle	−121	384	17.5	−98	376	14.6	−115 ± 125	323 ± 85	19.8 ± 20.3
Clockwise-curve walking	42	541	−4.4	73	514	−8.0	11 ± 72	347 ± 53	−1.8 ± 11.6
Counterclockwise-curve walking	−51	530	5.5	−147	492	16.6	14 ± 241	315 ± 74	−1.6 ± 37.8

## Data Availability

Publicly available datasets were analyzed in this study. This data can be found here: https://www.dropbox.com/s/jh1omfkuiuwf48j/VICON_data.zip?dl=0, accessed on 4 June 2021.
